# Oncogenic and tumor suppressor genes expression in myeloproliferative neoplasms: The hidden side of a complex pathology

**DOI:** 10.1002/jcla.24289

**Published:** 2022-02-17

**Authors:** Elham Abedi, Mehran Karimi, Ramin Yaghobi, Hamid Mohammadi, Sezaneh Haghpanah, Mohamad Moghadam, Elahe Bayat, Alireza Rezvani, Mani Ramzi

**Affiliations:** ^1^ 48435 Hematology Research Center Shiraz University of Medical Sciences Shiraz Iran; ^2^ 48435 Transplant Research Center Shiraz University of Medical Sciences Shiraz Iran; ^3^ Department of Pediatrics, School of Medicine Shiraz University of Medical Sciences Shiraz Iran; ^4^ 48435 Department of Biochemistry School of Medicine Shiraz University of Medical Sciences Shiraz Iran

**Keywords:** *ADAMTS18*, *BCR‐ABL1*, *CDKN2B*, *CMTM5*, *DCC*, *FHIT*, gene expression, myeloproliferative neoplasms, *WNT5B*

## Abstract

**Background:**

The present study aimed to explore the changes in the expressions of six tumor‐related genes in myeloproliferative neoplasms (MPNs). The study population included 130 patients with MPNs (52 with chronic myeloid leukemia (CML), 49 with essential thrombocythemia (ET), 20 with polycythemia vera (PV), and 9 with primary myelofibrosis (PMF)) and 51 healthy individuals.

**Methods:**

The expression profiling of six genes (*ADAMTS18*, *CMTM5*, *CDKN2B*, *DCC*, *FHIT*, and *WNT5B*) in the peripheral blood granulocyte cells was explored by real‐time quantitative reverse transcription polymerase chain reaction.

**Results:**

The patients with MPNs showed significant downregulation of *CMTM5* (EFC = 0.66) and *DCC* (EFC = 0.65) genes in contrast to a non‐significant upregulation of *ADAMTS18*, *CDKN2B*, *FHIT*, and *WNT5B* genes. Downregulation of *DCC* was consistent in all subtypes of MPN (EFC range: 0.591–0.860). However, *CMTM5* had a 1.22‐fold upregulation in PMF in contrast to downregulation in other MPN subtypes (EFC range: 0.599–0.775). The results revealed a significant downregulation in *CMTM5* and *DCC* at below 60‐years of age. Furthermore, female patients showed a clear‐cut downregulation in both *CMTM5* and *DCC* (EFC *DCC*: 0.436 and *CMTM5*: 0.570), while male patients presented a less prominent downregulation with a borderline *p*‐value only in *DCC* (EFC: 0.69; *p* = 0.05).

**Conclusions:**

Chronic myeloid leukemia cases showed a significant upregulation of *WNT5B*, as a known oncogenesis gene. Two tumor suppressor genes, namely *DCC* and *CMTM5*, were downregulated in the patients with MPNs, especially in females and patients below 60 years of age.

## Graphical abstract

The presence of the Philadelphia chromosome led to a significant upregulation of WNT5B with non‐significant changes in other genes. Two tumor suppressor genes, DCC and CMTM5, were downregulated in MPN patients especially in females and patients younger than 60 years old. WNT5B, a known oncogenesis gene, was upregulated in CML patients.

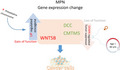

**Abbreviations**
CALR: calreticulin
CML: chronic myeloid leukemia
ET: essential thrombocythemia
JAK2: Janus kinase
MPN‐: *BCR*‐*ABL1*‐negative
MPN+: *BCR*‐*ABL1*‐positive
MPNs: myeloproliferative neoplasms
PMF: primary myelofibrosis
PV: polycythemia vera

## 1 INTRODUCTION

Myeloproliferative neoplasms (MPNs) refer to a heterogeneous group of hematologic disorders. According to the World Health Organization (WHO) (2016), these neoplasms have been divided into three main subgroups based on *JAK2*/*CALR*/*MPL* mutation; that is, Essential thrombocythemia (ET), polycythemia vera (PV), and primary myelofibrosis (PMF), and four clinicopathologic conditions including chronic myeloid leukemia (CML), chronic neutrophilic leukemia (CNL), chronic eosinophilic leukemia, and unclassifiable (MPN‐U).^1^, ^2^, ^3^, ^4^, ^5^, ^6^ ET, PV, and PMF are *BCR*‐*ABL1*‐negative MPNs, while chronic myeloid leukemia is a *BCR*‐*ABL1*‐positive MPN.^6^, ^7^ ET and PV are most often present with a rise in the platelet count and hemoglobin /hematocrit, respectively, both of which being accompanied by the risk of hemorrhage and vascular thrombosis. PMF is an advanced subtype of MPNs whose clinical presentations are remarkably more heterogeneous compared to ET and PV. PMF is associated with the release of fibrosis and pro‐inflammatory cytokines, bone marrow fibrosis, and often extensive extramedullary hematopoiesis in the spleen or liver. CML is caused by the acquisition of *BCR*‐*ABL1* in hematopoietic stem cells, which transforms them into leukemic stem cells (LSC). *BCR*‐*ABL1* mutation results from a reciprocal translocation between the long arms of chromosomes 9 and 22, leading chromosome 22 to become shorter.^8^, ^9^ Molecular monitoring of *BCR*‐*ABL1* for CML using the international scale (IS) has become the model for molecular monitoring of other types of leukemia and diseases.^10^, ^11^

Since the discovery of *Jak2V617* followed by *CALR* and other mutations in patients with MPNs, a great number of genomic studies have revealed more somatic alterations in the majority of these patients. Although the mutational events involved in MPNs pathogenesis have been comprehensively determined, the impact of different somatic alterations on gene expression and transcriptional output has not been evaluated yet. In the MPNs family, especially in CML, some downstream signaling pathways account for the progression of the disease. This factor alongside molecular events alters the expression profiles of several important genes that may play a crucial role in the evolution and pathogenesis of MPNs. Therefore, the present study aimed to assess the expression levels of a number of genes associated with DNA methylation, as the common epigenetic change in hematological cancers. For this purpose, the raw data from GeoDataSet NCBI (GSE87806) and the previous studies were reviewed to identify the most likely genes involved in this process. Accordingly, *ADAMTS18*, *CMTM5*, *CDKN2B*, *FHIT*, WNT5B, and *DCC* were among the genes more commonly affected by methylation changes in MPNs.^12^, ^13^, ^14^, ^15^, ^16^, ^17^, ^18^, ^19^

## 2 MATERIALS AND METHODS

In this cross‐sectional study, patients with MPNs referred to the hematology‐oncology department of Namazee Hospital, Shiraz, Iran from May 2018 to May 2019 were selected as the patient group. A group of age‐ and sex‐matched volunteers was also selected as the control group. The patients were diagnosed based on the WHO’s criteria and clinical, laboratory, and molecular analyses. Written informed consent was obtained from all the participants. The study design was approved by the Ethics Committee of Shiraz University of Medical Sciences, Shiraz, Iran (ethics code: IR.SUMS.REC1397.535).

### 2.1 Total RNA extraction and cDNA synthesis

Peripheral blood granulocyte cells were collected from the patient and control groups by density gradient methods using lymphodex (Inno‐train). Total RNA was extracted using RiboExTM (GeneAll) according to the manufacturer's instructions. The quantity and quality of the extracted RNA were measured using the Nano‐Drop ND‐1000 spectrophotometer (Thermo Fisher Scientific Inc). RNA integrity was also evaluated using electrophoresis on 1% agarose gel. Besides, the synthesis of complementary DNA (cDNA) was done using Prime RT Premix cDNA synthesis kit (Genet Bio) according to the manufacturer's instructions. The samples were stored at −70°C.

### 2.2 *ADAMTS18*, *CMTM5*, *CDKN2B*, *DCC*, *FHIT*, and *WNT5B* mRNA expression

Specific primers for HPRT1 (as the internal control or housekeeping gene) and *ADAMTS18*, *CMTM5*, *CDKN2B*, *DCC*, *FHIT*, and *WNT5B* genes were designed using the AlleleID software (PREMIER). To prevent the genomic DNA amplification exon/exon junction spanning primers or Intron interval primers were designed. (the details regarding the quantitative reverse transcription polymerase chain reaction (qRT‐PCR) primers have been given in Table 1. The primers were blasted with the entire human genome. The primers used for RT‐PCR for all gene amplifications were synthesized by Metabion, Germany (Table 1).

**TABLE 1:** Primer sequences and PCR product sizes of various genes studied

	Primer sequence	Amplifier size	Accession No.
*WNT5B*	F:GCAGCACAGCGGACAACG R:CGTGGGTGAAGGCGGTCTC	75	NM_032642.3
*ADAMTS18*	F:AGCCCAAGCAAGCAGGACAGTA R:GCGGGCATAAACTTGGTCTCACA	190	NM_199355.4
*CMTM5*	F:AAACCGAGCTGGCCCTGAC R:AAGAGGAAGGCAAGTGTGTGATGAA	109	NM_001288746.2
*CDKN2B*	F:CGGAGGTCATGATGATGG R:GGTCGGGTGAGAGTGGCA	97	NM_004936.4
*DCC*	F:AGCCCAGCAGAGAAAGAAAC R:GGTGTGAGGTCTTGGCAACT	186	NM_005215.4
*HPRT1*	F:GGCGTCGTGATTAGTGATGATGA R:ACCCTTTCCAAATCCTCAGCATAA	86	NM_000194.3
*FHIT*	F:GGAATACCTGCCTGCTTAGA R:ACAAGAGCGAAGGACAGTT	179	NM_002012.4

### 2.3 Real‐time quantitative reverse transcription‐polymerase chain reaction

The relative expression profiles of *ADAMTS18*, *CMTM5*, *CDKN2B*, *DCC*, *HPRT1*, *FHIT*, and *WNT5B* genes were assessed by SYBR Green I real‐time PCR Chemistry, with the *HPRT1* gene being used as the endogenous control gene. Briefly, PCR reactions were performed in a final volume of 10 μl containing 1 μl of the cDNA, 5 μl of 2X Bio fact SYBR Master Mix (Daejeon), and 0.5 pM of the primer pairs. After initial denaturation at 95°C for 3 min, 40 cycles consisting of the following steps were performed using IQ5 Real‐Time PCR System (BIO‐RAD the US): 30 seconds at 95°C, 30 s at 60°C for *FHIT*, 61°C for *CDKN2B*, *DCC*, and *HPRT*1, and 59°C for *WNT5B*, *ADAMTS18*, and *CMTM5* genes as the annealing step, and 20 s at 72°C. Each PCR reaction was done in triplicates. After completing the polymerase cycle, a melting curve analysis was performed to identify the non‐specific PCR products and primer dimer formation. The relative expression data of the genes relative to the internal control gene was obtained using the 2^(−ΔΔCT)^ method (Livak method). The fold changes were further converted to the log 10 scale. Then, the mean values of the relative fold changes in the patient group and healthy controls were calculated, analyzed, and compared.

### 2.4 Statistical analysis

The value of ΔCts was analyzed for normality of distribution by the Shapiro–Wilk test. For normally distributed data, mean and SD were calculated. Additionally, analysis of variance was used to compare different groups under investigation. Non‐normally distributed data were analyzed by non‐parametric tests, and medians and ranges were compared using Mann–Whitney test. All data analyses were carried out using the SPSS 18 software and *p* < 0.05 was considered statistically significant.

## 3 RESULTS

This study was conducted on 130 patients with MPNs including 52 patients with positive *BCR*‐*ABL1* mutation and 78 with negative *BCR*‐*ABL1* mutation (49 with ET, 20 with PV, and 9 with PMF) along with 51 healthy individuals. The mean age of the participants was 53.2 ± 15 years in the patient group and 48.8 ± 16.4 years in the control group (*p* = 0.054). The mean age of the patients with PV was 63.4 ± 13.9 years, which was significantly higher compared to other MPN categories and the control group (*p* = 0.021). The male to female ratio was equal to one in the patient group (Table 2).

**TABLE 2:** Main clinical and hematological features of 130 MPN patients and healthy control

Groups	CML (*n* = 52)	PV (*n* = 20)	ET (*n* = 49)	PMF (*n* = 9)	Healthy controls (*n* = 51)
Mean age* (year ± SD)	49.7 ± 12.8	63.4 ± 13.9	52 ± 15	56.7 ± 20.2	48.8 ± 16.4
Sex, (male/female)	24/28	12/8	24/25	5/4	26/25
Mean WBC count (×10^3^)	35.6 ± 21.6	18.5 ± 8.9	14.9 ± 11.3	11.5 ± 9.8	7.6 ± 2.8
Mean Hb level (gr/dl)	11.6 ± 3.4	17.6 ± 5.2	12.3 ± 6.4	12.1 ± 4.8	12.4 ± 2.1
Mean platelet count (×10^3^)	390.6 ± 112.8	488.2 ± 156.4	690.7 ± 321.7	365.7 ± 196.3	187.3 ± 48.9
Splenomegaly	21/52	7/20	11/49	4/9	‐

Abbreviations: CML, chronic myeloid leukemia; ET, essential thrombocythemia; MPNs, myeloproliferative neoplasms; PMF, primary myelofibrosis; PV, polycythemia vera.

* *p* = 0.09 between the patient and control groups (no significant difference). However, a significant difference was found between the patients with PV and other patient groups in terms of mean age (*p* = 0.004).

### 3.1 Gene expression

The results showed the normal ΔCts distribution of *CMTM5*, *DCC*, and *FHIT* (Shapiro–Wilk test: 0.290, 0.291, and 0.159, respectively) in contrast to the non‐normal ΔCts distribution of *ADAMTS18*, *WNT5B*, and *CDKN2B* (Shapiro–Wilk test: 0.027, 0.001, and 0.005, respectively). In the following sections, the data have been presented as mean ± SD for the normally distributed data and as median (range) or box‐plot chart for the non‐normally distributed data. *p*‐values have been calculated based on the data type (please refer to the statistics section). Expression fold change (EFC) shows the extent of downregulation (values less than one) and upregulation (values more than one).

Gene expression profiles of *ADAMTS18*, *CMTM5*, *CDKN2B*, *DCC*, *FHIT*, and *WNT5B* mRNAs in all the patients and controls have been presented in Table 3. The results revealed a significant downregulation in *CMTM5* (EFC = 0.66) and *DCC* (EFC = 0.65) genes and a non‐significant upregulation of *ADAMTS18*, *CDKN2B*, *FHIT*, and *WNT5B* genes compared to the control group.

**TABLE 3:** Comparison of the patient and control groups regarding the gene expression fold changes

Non‐normally distributed data presented as median and compared using Mann–Whitney test							
		*N*	Median delta CT			*p*‐Value	Expression fold change
			Median	Min	Max		
*ADAMTS18*	Patient	130	3.37	0.68	6.48	0.213	1.222
	Control	51	3.85	0.53	6.70		
	Total	181	3.50	0.53	6.70		
*WNT5B*	Patient	130	2.90	−1.17	6.70	0.376	1.218
	Control	51	3.26	−0.68	5.53		
	Total	181	3.07	−1.17	6.70		
*CDKN2B*	Patient	130	3.34	0.92	6.75	0.784	1.044
	Control	51	3.87	1.03	6.41		
	Total	181	3.54	0.92	6.75		

Normally distributed data presented as mean delta CT ± SD					
		*N*	Mean delta CT ± SD	*p*‐Value	Expression fold change
*CMTM5*	Patient	130	−2.915 ± 1.194	**0.003**	0.666
	Control	51	−3.501 ± 1.061		
	Total	181	−3.080 ± 1.1853		
*DCC*	Patient	130	4.662 ± 1.227	**0.002**	0.652
	Control	51	4.0451 ± 1.11133		
	Total	181	4.488 ± 1.225		
*FHIT*	Patient	130	−1.381 ± 1.480	0.488	1.120
	Control	51	−1.216 ± 1.282		
	Total	181	−1.334 ± 1.425		

Bold indicates significant *p* value (<0.05).

**FIGURE 1:**
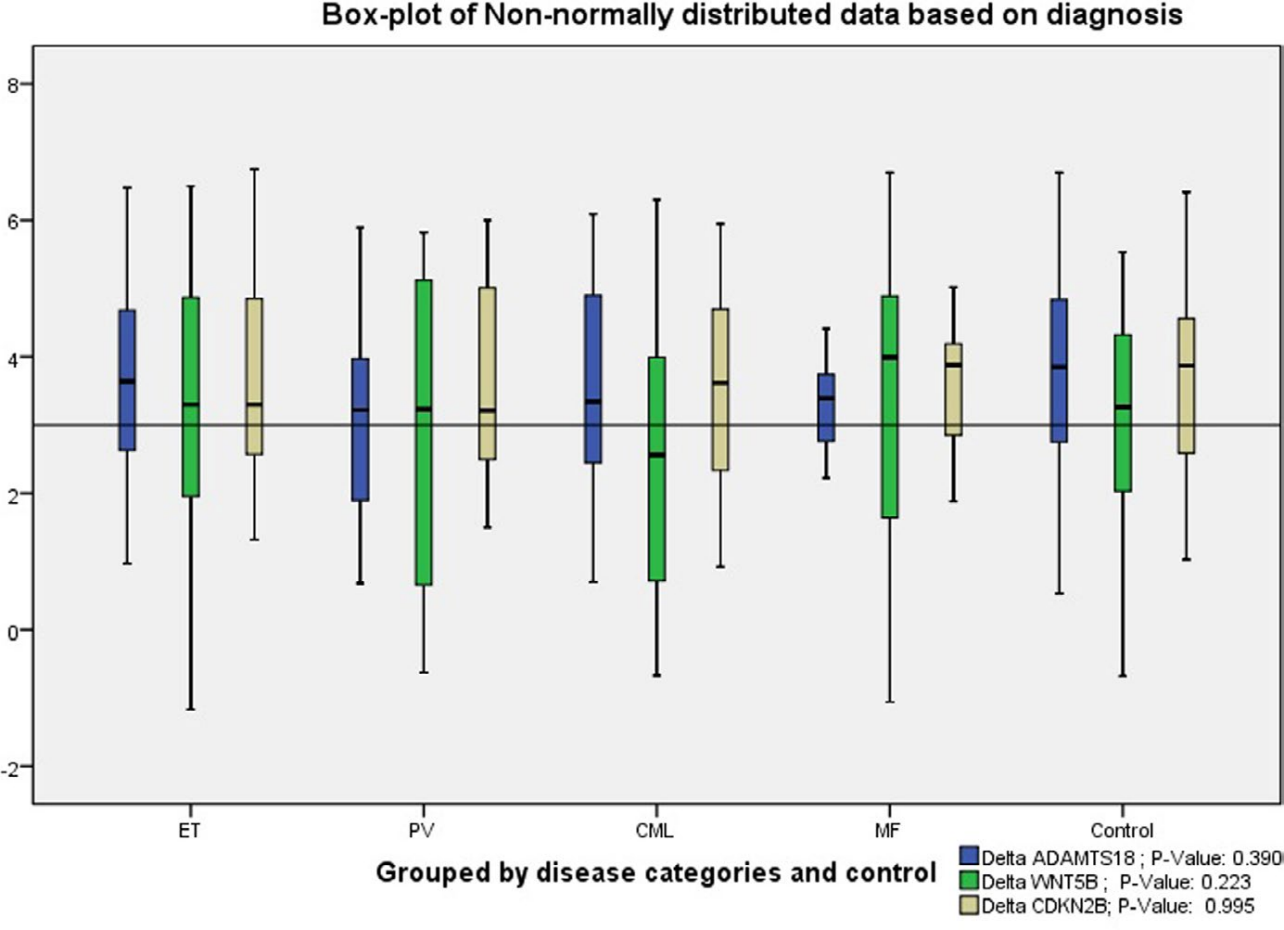
Box‐plot chart of delta CT in *ADAMTS18*, *WNT5B*, and *CDKN2B* in patient and control groups. The color legend includes a *p*‐value between the categories based on Mann–Whitney *U*‐test. MPNs, myeloproliferative neoplasms (*n* = 130); CML, chronic myeloid leukemia (*n* = 52); ET, essential thrombocythemia (*n* = 49); PMF, primary myelofibrosis (*n* = 9); PV, polycythemia vera (*n* = 20)

Detailed analysis of the expression changes has been presented in Table 4 (parametric data) and Figure 1 (non‐parametric data). The results indicated the significant downregulation of *DCC* expression in all the subtypes of MPN compared to the control group. Interestingly, *CMTM5* expression showed a 1.22‐fold upregulation in the patients with PMF compared to other MPN categories, and the difference was statistically significant. However, it presented no significant upregulation compared to the control group. No significant difference was observed among the MPN categories with respect to the expression of other genes including *ADAMTS18*, *CDKN2B*, *FHIT*, and *WNT5B* (Table 4 and Figure 1).

**TABLE 4:** Gene expression changes in different MPN categories

	*N*	Mean	Std. deviation	Expression fold change	*p*‐Value
*CMTM5*	ET	49	−2.761 ± 1.080	0.599	0.003
	PV	20	−3.134 ± 1.277	0.775	
	CML	52	−2.823 ± 1.235	0.625	
	MF	9	−3.798 ± 1.088	1.229	
	Control	51	−3.501 ± 1.061		
	Total	181	−3.080 ± 1.185		
*DCC*	ET	49	4.604 ± 1.163	0.678	0.024
	PV	20	4.619 ± 1.428	0.672	
	CML	52	4.802 ± 1.178	0.591	
	MF	9	4.262 ± 1.465	0.860	
	Control	51	4.045 ± 1.111		
	Total	181	4.488 ± 1.225		
*FHIT*	ET	49	−1.244 ± 1.500	1.019	0.835
	PV	20	−1.431 ± 1.767	1.160	
	CML	52	−1.436 ± 1.437	1.164	
	MF	9	−1.693 ± 1.003	1.391	
	Control	51	−1.216 ± 1.282		
	Total	181	−1.334 ± 1.425		

In the next step, we tried to explore the relationship between gene expression and age and sex Tables 5 and 6. In this study, the participants were divided into lower and higher than 60 years age groups and were compared concerning gene expression. The results revealed no significant difference in this regard in the patient and control groups. However, a statistically significant difference was found in the means of delta *CMTM5* and delta *DCC* between the individuals younger than 60 years in the MPN and control groups (Table 5). In the individuals aged below 60 years, both genes showed downregulation in the patients with MPNs (EFC = 0.66 and 0.64 for *CMTM5* and *DCC* genes, respectively) (Table 5). Nevertheless, the results demonstrated no significant difference between the two age categories in the patient and control groups regarding *ADAMTS18*, *CDKN2B*, *FHIT*, and *WNT5B* (Table 5).

**TABLE 5:** Comparison of the two age categories regarding the mean delta CT

Non‐parametric factor		*N*	Delta CT in the MPN group			*p*‐Value in the MPN group	Delta CT in the control group			EFC	*p*‐Value between the MPN and control groups
			Median	Min	Max		Median	Min	Max		
*ADAMTS18*	Age <60 years	86	3.13	0.70	6.48	0.379	3.77	0.53	6.70	1.252	0.249
	Age ≥60 years	44	3.38	0.68	6.29		4.13	1.26	5.37	1.176	0.576
	Total	130	3.37	0.68	6.48		3.85	0.53	6.70	1.222	
*WNT5B*	Age <60 years	86	2.76	−1.17	6.31	0.414	3.37	−.68	5.53	1.370	0.221
	Age ≥60 years	44	3.25	−0.67	6.70		3.04	0.43	5.33	0.948	0.903
	Total	130	2.90	−1.17	6.70		3.26	−.68	5.53	1.218	
*CDKN2B*	Age <60 years	86	3.53	1.32	6.75	0.372	3.67	1.03	6.41	0.919	0.652
	Age ≥60 years	44	3.18	0.92	5.95		4.24	1.15	6.24	1.383	0.263
	Total	130	3.34	0.92	6.75		3.87	1.03	6.41	1.044	

Parametric factors		MPN number	Delta CT in the MPN group (Mean ± SD)	*p*‐Value in the MPN group	Mean of delta CT in the control group	EFC	*p*‐Value between the MPN and control groups
CMTM5	Age <60 years	86	**−2.940 ± 1.095**	**0.740**	**−3.543**	0.659	0.007
	Age ≥60 years	44	−2.866 ± 1.380		−3.401	0.690	0.165
	Total	130	−2.915 ± 1.194		−3.501	0.666	
DCC	Age <60 years	86	**4.676 ± 1.191**	0.855	**4.033**	**0.640**	**0.007**
	Age ≥60 years	44	4.634 ± 1.310		4.073	0.678	0.143
	Total	130	4.662 ± 1.227		4.045	0.652	
FHIT	Age <60 years	86	−1.358 ± 1.447	0.807	−1.408	0.965	0.856
	Age ≥60 years	44	−1.425 ± 1.559		−.7560	1.591	0.135
	Total	130	−1.381 ± 1.480		−1.216	1.120	

Abbreviation: EFC, expression fold change.

Bold indicates significant *p* value (<0.05).

**TABLE 6:** Comparison of males and females regarding gene expression

		Male						Female					
		*N*	Delta CT			*p*‐Value in males	EFC	*N*	Delta CT			*p*‐Value in females	EFC
Non‐parametric factors			Median	Min	Max				Median	Min	Max		
*ADAMTS18*	Patient	65	3.32	.68	6.48	0.172	1.32	65	3.39	.87	5.47	0.753	0.99
	Control	34	4.04	.53	6.20			17	3.47	1.38	6.70		
	Total	99	3.66	.53	6.48			82	3.43	.87	6.70		
*WNT5B*	Patient	65	2.83	−1.06	6.70	0.457	1.28	65	3.13	−1.17	6.22	0.933	1.07
	Control	34	3.26	.43	5.53			17	3.10	−.68	5.03		
	Total	99	3.01	−1.06	6.70			82	3.12	−1.17	6.22		
*CDKN2B*	Patient	65	3.62	.92	6.75	0.763	1.04	65	3.21	1.50	5.83	0.834	0.96
	Control	34	3.77	1.03	6.41			17	3.87	1.15	5.25		
	Total	99	3.66	.92	6.75			82	3.27	1.15	5.83		

Parametric factors		*N*	Delta CT (mean ± SD)	*p*‐Value	EFC	N	Delta CT (mean ± SD)	*p*‐Value	EFC
*CMTM5*	Patient	65	−3.034 ± 1.288	0.39	0.858	65	−2.79 ± 1.08	**0.001**	0.436
	Control	34	−3.255 ± 1.052			17	−3.992 ±.92		
	Total	99	−3.110 ± 1.212			82	−3.044 ± 1.158		
*DCC*	Patient	65	4.657 ± 1.263	**0.05**	0.698	65	4.667 ± 1.2012	**0.015**	0.570
	Control	34	4.139 ± 1.168			17	3.857 ± 0.993		
	Total	99	4.479 ± 1.250			82	4.499 ± 1.201		
*FHIT*	Patient	65	−1.229 ± 1.608	0.80	1.055	65	−1.532 ± 1.336	0.612	1.138
	Control	34	−1.152 ± 1.245			17	−1.346 ± 1.381		
	Total	99	−1.202 ± 1.487			82	−1.494 ± 1.339		

Abbreviation: EFC, expression fold change.

Bold indicates significant *p* value (<0.05).

A comparison of the sex‐related gene expression in the study population has been depicted in Table 6. The male and female participants in the control and MPN groups were separately compared with regard to delta CT. The results only showed a borderline difference between the male participants in the control and MPN groups regarding delta *DCC* (EFC = 0.69, *p* = 0.05). On the other hand, the results indicated a significant downregulation in the expressions of both *CMTM5* and *DCC* genes in the female patients compared to the controls (Table 6).

**FIGURE 2:**
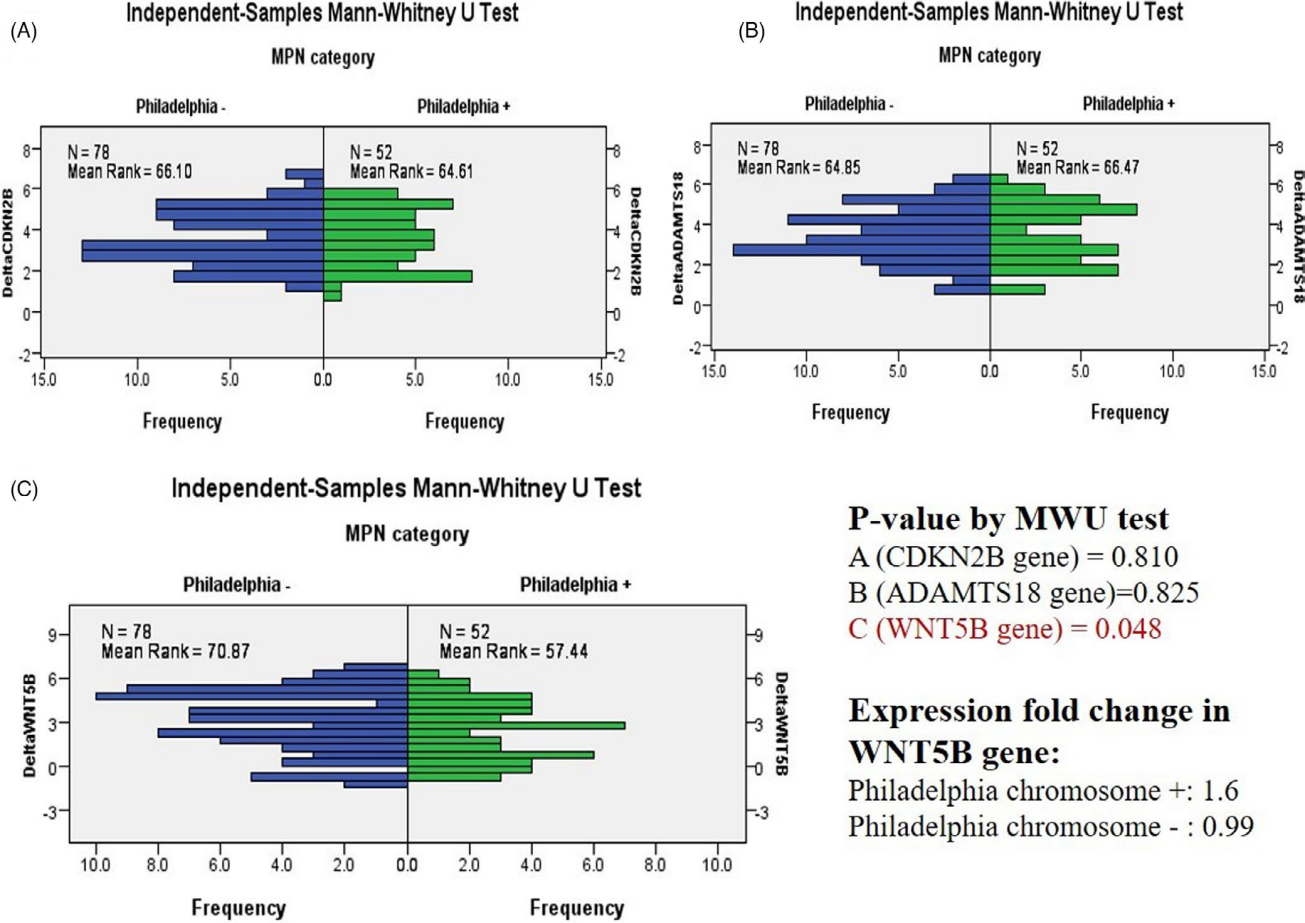
Delta CT comparison by Mann–Whitney test showed a significant difference between the patients with (*n* = 52) and without (*n* = 78) the Philadelphia chromosome. The patients with the Philadelphia chromosome presented the upregulation of the WNT5B gene (*p* = 0.048)

The *BCR*‐*ABL1* mutation effect has been shown in Table 7 (normally distributed data) and Figure 2 (non‐normal distributed data). The positive *BCR*‐*ABL1* mutation led to the overexpression of the *WNT5B* gene (EFC = 1.6, *p* = 0.048). However, no significant difference was detected among other genes regarding the *BCR*‐*ABL1* mutation status.

**TABLE 7:** Comparison of the mean gene expression in the patients with and without the *BCR*‐*ABL1* mutation

Parametric factor	*BCR*‐*ABL1* chromosome	*N*	Mean delta CT ± SD		*p*‐Value	Expression fold
*CMTM5*	*BCR*‐*ABL1*−	78	−2.976 ± 1.169		0.475	0.695
	*BCR*‐*ABL1*+	52	−2.823 ± 1.235			0.625
	Total	130	−2.915 ± 1.194			0.666
*DCC*	*BCR*‐*ABL1*−	78	4.568 ± 1.258		0.289	0.696
	*BCR*‐*ABL1*+	52	4.802 ± 1.178			0.591
	Total	130	4.662 ± 1.227			0.652
*FHIT*	*BCR*‐*ABL1*−	78	−1.344 ± 1.517		0.728	1.092
	*BCR*‐*ABL1*+	52	−1.436 ± 1.437			1.164
	Total	130	−1.381	1.480		1.120

## 4 DISCUSSION

According to the 2016 classification system of hematologic malignancies, based on the *BCR*‐*ABL1* mutation, MPNs can be divided into two groups; that is, *BCR*‐*ABL1*+ (CML) and *BCR*‐*ABL1*‐ (PV, ET, and PMF). DNA methylation, the most common epigenetic change, plays a significant role in the pathogenesis of different hematologic malignancies including MPNs. Totally, two abnormal methylation patterns have been found in some cancers, resulting in a decrease or an increase in transcription. Although the exact mechanism leading to abnormal DNA methylation is unknown, chronic inflammation, oxidative stress, alcohol, and aging have been claimed to contribute to the process.^8^, ^17^, ^20^ Evidence has indicated that heterogeneous genes are affected in this process. After deeply investigating the previous studies and the GEO databases, the six genes presented in the current study (five tumor suppressors and one oncogene) were found to be more likely to be affected by the methylation process. In the present study, therefore, the ECF of the selected genes was explored as the first step to explore the possible association between these genes and MPN pathogenesis and clinical outcome.^7^, ^16^, ^17^, ^21^, ^22^ To the best of our knowledge, the present study is one of the few studies exploring the expression levels of some tumor suppressors and oncogenes in a population of patients with MPNs.

Among the five genes mainly known as tumor suppressor genes, two (*CMTM5* and *DCC*) showed a significant downregulation. These genes are involved in many biological processes in the human body. For instance, *CMTM5* has a tumor inhibitory function, an immune system modulation capability, and an active role in the male reproductive system.^16^, ^22^ Many cancers such as myeloid leukemia, prostate cancer, and cervical cancer are associated with the downregulation of this gene.^16^, ^22^, ^23^ Another tumor suppressor gene, which is primarily known as “deleted in colorectal carcinoma” or *DCC*, encodes the netrin receptor protein and acts as both a conditional oncogene and a conditional tumor suppressor.^24^, ^25^ After the initial detection of this gene in colorectal cancer, its role has been rapidly highlighted in other malignancies. To date, the effect of this gene has been recognized in many malignancies including hematologic malignancies. The absence of *DCC* is a prognostic factor in AML and MDS pathogenesis. Additionally, tumor suppression and metastasis suppression have been reported with the restitution of normal *DCC* function. Inokuchi et al. disclosed that the absence of the *DCC* gene contributed to the pathogenesis of MDS and AML and might worsen the AML prognosis.^25^, ^26^ In another study on a rare form of MPN; that is, chronic neutrophilic leukemia, the role of *DCC* was investigated in the MPN subcategories. The results demonstrated that *DCC* heterozygote patients had a shorter latency period compared to those with the myeloproliferative disease.^27^ Furthermore, less than 30% *DCC* gene absence or downregulation was reported in CML cases.^28^, ^29^ In colorectal cancer, the absence or downregulation of this gene was associated with poor prognosis and an increased rate of metastasis and progression from the benign to a malignant form of colorectal tumors. It also played an important role in the loss of homogeneity, which contributed to poor prognosis in these tumors.^30^, ^31^, ^32^, ^33^ Up to now, little attention has been paid to the role of this gene and its expression. Considering the 0.652 *DCC* downregulation among the cases with MPNs (Table 3) that was significant compared to the control group (*p* = 0.002), the influence of this gene and its related functions is suggested to be taken into account in some pathogeneses and phenotypic presentations of MPNs.

The role of *CM5MT* in hematological and non‐hematological malignancies has been investigated in different studies, indicating this gene as a potent tumor suppressor gene.^16^, ^22^, ^23^, ^34^, ^35^ This gene encodes a member of the chemokine‐like factor superfamily and exhibits tumor suppressor features. Niu Jihong et al. reported the downregulation of *CMT5 M* in the AML cases and its return to the normal level with successful treatment.^22^ The main role of *CMT5 M* in malignancies is related to the induction of apoptosis and cell‐cycle arrest. Therefore, its down‐regulation leads to the survival of malignant cells.^35^, ^37^, ^38^ The present study findings showed the significant downregulation (near 40%) of this gene in MPNs, which is probably a contributing factor to the pathogenesis of this proliferative neoplasm. Yet, more precise investigations are required due to the multifactorial nature of this category of hematological malignancies.

The current study results revealed the upregulation of four genes including *ADAMTS18*, *CDKN2B*, and *FHIT* as tumor suppressors and *WNT5B* as an oncogene in the patients. However, no significant difference was found in the control group in this regard. Although these genes have multiple functions, they all have tumor suppressor activities with different mechanisms.^39^, ^40^, ^41^, ^42^ Their normal functions decrease their role in the pathogenesis of MPNs.

In the current research, the subcategories of MPNs were reanalyzed to obtain more details about the downregulation of these genes. Although the PMF cases had a lower *DCC* EFC, all the subcategories of MPNs showed downregulations with EFCs ranging from 0.6 to 0.8 (Table 4). The *CMMT5* gene had a different pattern of EFC among the MPN subcategories. This gene was downregulated in the patients with ET, PV, and CML, but upregulated in those with PMF. No similar studies were found to compare the results obtained in the patients with PMF. Hence, explaining this finding needs further investigations, and the results should be interpreted in the context of PMF pathophysiology and the wide range of *CMTM5* functions such as the immune system. Age is one of the possible factors in changes in gene expression. Thus, the EFC was analyzed in two age categories. Interestingly, the results indicated that both *DCC* and *CMTM5* genes were significantly downregulated in the patients aged below 60 years compared to the age‐matched controls. This implied that the downregulation of *DCC* and *CMTM5* played a more important role in the pathogenesis of MPNs at ages below 60 years. However, the difference between the two age groups was on the borderline and, consequently, should be reinvestigated in future studies with larger sample sizes (Table 5). Some other studies in different contexts and malignancies did not report any correlation between age and the gene expression of *CMTM5*.^43^ As expected, the present study findings also showed the ineffectiveness of age in the gene expressions of *ADAMTS18*, *CDKN2B*, *FHIT*, and *WNT5B*.

Furthermore, the role of sex in the expression of these genes was evaluated in this study. Based on the results, females showed a more prominent downregulation of *DCC* and *CMTM5* in the setting of MPNs (Table 6). However, none of these genes had a significantly different expression between the males and females in the control group. Thus, the sex difference in the expression rate was ruled out. The role of the female sex was more prominent in the *CMTM5* gene expression. In other words, in the female patients with MPNs, the maximum downregulation among all the genes was observed for *CMTM5* with EFC = 0.436. Nonetheless, the male patients showed no significant difference in the expression of this gene (male ECF =0.858; *p*‐value with the control group =0.36). This significant female predominance in *CMTM5* downregulation was not suggested by other studies on *CMTM5* expression in hematological or non‐hematological malignancies.^22^, ^23^, ^43^

Considering the *DCC* gene, the expression change was significant in the female patients. Male patients also showed a borderline significant downregulation compared to the male controls (male EFC = 0.698, *p* = 0.05; female EFC = 0.570, *p* = 0.015). This highlighted the role of *CMTM5* in the pathogenesis of MPN, particularly in female patients.

In the current investigation, the CML patients presented the overexpression of the *WNT5B* gene (*p* = 0.048). Considering the non‐normal distribution of the data represented in Figure 2, Mann–Whitney test was employed. The results revealed that EFC was close to one among the patients with negative *BCR*‐*ABL1* mutation, which implied no change in gene expression. Nevertheless, the positive cases had the highest value of overexpression (EFC = 1.6). It seemed that the *BCR*‐*ABL1* mutation had a synergistic effect on the expression of the *WNT5B* gene. The overexpression of this gene, as an oncogene, has been reported in many studies, some of which indicated *BCR*‐*ABL*+as an aggravating factor for its overexpression.^44^, ^45^ This gene could promote cell migration and invasion, eventually leading to poor outcomes^17^, ^46^ thus, it may be reasonable to investigate the role of *WNT5B* overexpression in the prognosis of *BCR*‐*ABL1*+ cases.

## 5 CONCLUSION

The present study was one of the few studies investigating the role of tumor suppressors and oncogenes in MPNs. MPNs and their subcategories exhibited significant *CMTM5* and *DCC* downregulations, which were pronounced in females as well as in individuals aged below 60 years. Additionally, the *WNT5B* oncogene was overexpressed in the CML cases (*BCR*‐*ABL1*+ cases). Yet, further clinical studies are required to be conducted on EFC in tumor suppressors and oncogenes so as to provide new insights into the pathogenesis of patients with MPNs.

## CONFLICT OF INTEREST

None declared.

## INFORMED CONSENT

The study was explained to the patients and their informed consent forms were obtained.

## DECLARATION

To the best of our knowledge, the present study is one of the few studies exploring tumor suppressor genes or oncogenes in a population of MPN patients (Philadelphia positive & Philadelphia negative). This study aimed to evaluate the expression of multiple genes (five tumor suppressors and one oncogene) and to highlight the relationship between these genes and some patients’ features. Two tumor suppressor genes, *CMTM5* and *DCC*, were downregulated in patients with MPN, especially in females and younger cases. Known oncogenesis, WNT5B, is overexpressed by up to 1.6 folds in Philadelphia positive MPNs. The findings pertaining to oncogenes and tumor suppressors may illuminate a new pathway in the MPN pathogenesis or a new index for MPN treatment and outcome.

## Data Availability

The author elects to not share data.
